# Pulpal calcifications in orthodontically moved teeth: Scoping review

**DOI:** 10.4317/jced.60777

**Published:** 2023-09-01

**Authors:** Zilda Farias, Juliana Sousa, Caroline Faria, Juliana Vieira, Ana Sobral, Marcia Silveira

**Affiliations:** 1 PhD student, Department of Oral and Maxillofacial Pathology, School of Dentistry, University of Pernambuco (UPE), Recife, Brazil; 2 Associate Professor, Department of Oral and Maxillofacial Pathology, School of Dentistry, University of Pernambuco (UPE), Recife, Brazil

## Abstract

**Background:**

The application of forces during orthodontic treatment can induce pulpal calcifications, characterized by the deposition of mineralized tissue in the pulp cavity space, there may be repercussions on dental procedures, especially endodontic treatment. The objective of this article is to map the scientific evidence and any gaps in knowledge regarding the relationship between orthodontics and dental pulp calcifications.

**Material and Methods:**

The study comprised a scoping review whose guiding question was: “What is the scientific evidence of the association between pulpal calcifications and orthodontic treatment?” Two independent reviewers searched the PubMed, Scopus, and Web of Science databases and the grey literature. Original articles and observational and clinical trials, which addressed the repercussions on the pulp cavity of teeth submitted to orthodontic treatment, were included. There was no language restriction or limitation of the year of publication until October 2022.

**Results:**

After critical reading and applying the eligibility criteria, 11 studies were included in this scoping review: six observational and five experimental studies. A statistically significant association of orthodontic treatment with changes in pulp cavity volume and increased incidence of pulp stones was observed among the studies findings.

**Conclusions:**

The orthodontic force can promote changes in the dental pulp that may cause direct implications in other dental treatments, especially endodontic treatment.

** Key words:**Dental pulp calcifications, pulp node, pulp obliteration, orthodontic treatment, orthodontics.

## Introduction

Upon masticatory or traumatic stimuli, the dental pulp may undergo changes in its vascular, neural, and cellular components in order to adapt (1). As a defense mechanism, the pulp response will vary according to the intensity, magnitude, and duration of the stimulus and may result in the formation of mineralized tissue (1,2).

The application of forces during orthodontic treatment can induce pulpal calcifications, characterized by the deposition of mineralized tissue in the pulp cavity space (3,4). The two main morphologies of pulpal calcifications include pulpal stones or nodes and diffuse calcifications.5 Other factors known to contribute to the formation of pulpal calcifications include dental trauma, bruxism, carious lesions, occlusal imbalance, and increasing age (2,5,6).

The presence of pulpal calcifications in healthy teeth does not bring great clinical relevance. However, when it comes to teeth with an indication for endodontic treatment, it deserves special attention. The obliteration of the pulp cavity, or the presence of pulp nodes, represents challenges for locating, accessing, and instrumenting root canals. Accidents such as instrument deflection and fracture can happen, and patency is often not possible during biomechanical preparation (3,5).

Proper and individualized planning is essential for endodontic treatment. Thus, it is of utmost importance to investigate the presence of pulpal calcifications before intervention in patients with a history of orthodontic treatment (3). Therefore, this scoping review aims to map the scientific evidence and possible gaps in knowledge regarding the relationship between orthodontics and dental pulp calcifications. Our hypothesis states that pulpal calcifications are present in teeth after orthodontic movement.

## Material and Methods

-Study design

This study comprised a scoping review aimed at mapping the literature on pulpal calcifications associated with orthodontic treatment. It was conducted according to the recommendations of the Joanna Briggs Institute Manual (JBI), Reviewers Manual 2020 (7) and the PRISMA Extension for Scoping Reviews (PRISMA-ScR) checklist (8). The research protocol for this review was registered with the Open Science Framework on October 24, 2022, and is available on osf.io/zr326.

-Identification of the research question or guiding question

The review’s guiding question was formulated according to the acronym participants, concept, and context (PCC), which stood for: P (Population) - patients undergoing orthodontic treatment; C (Concept) - dental pulp calcifications; and C (Context) - orthodontic movement. According to the proposed strategy, the research question asked: “What is the scientific evidence of the association between pulpal calcifications and orthodontic treatment?”

-Identification of relevant studies

In order to conduct this step, search strategies were developed. Then, in October 2022, two reviewers (J.S.S.S.S. and Z.B.B.M.F.) conducted independent searches using the PubMed, Scopus, and Web of Science databases. Furthermore, they searched the grey literature of non-indexed materials and the references of eligible articles. The search strategies were adapted according to the specificities of each database, maintaining the similarity of the combination of descriptors through Boolean operators (OR and AND). The Table 1 shows the descriptors and search strategy used in the research.

**Table 1:**
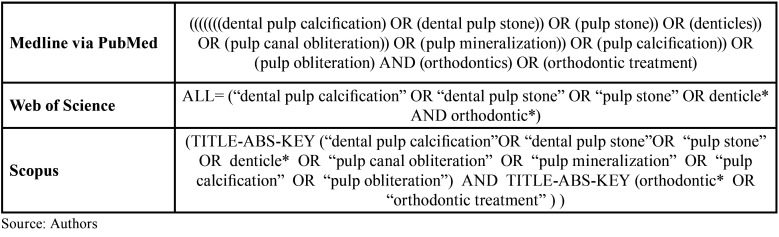
Search strategy.

Original articles and observational and clinical trials, which addressed the repercussions on the pulp cavity of teeth submitted to orthodontic treatment, were included. There was no language restriction or limitation of the year of publication until October 2022. Internet texts, editorials, experience reports, studies dealing with orthopedics, studies including patients with dysplasia and systemic diseases, studies involving dental trauma, review articles, and texts not available in full in the databases were excluded.

-Study selection

Two reviewers (J.S.S.S.S. and Z.B.B.M.F.) independently selected the studies in two stages. In the first stage, the articles were selected according to their titles and abstracts. In the second stage, the full texts of the previously identified articles were obtained, and those that met the eligibility criteria were selected by reading the full text.

Data analysis, grouping, synthesis, and presentation

A database was created based on the studies selected in the database search. The Endnote software was used to manage the references, including removing duplicates. The following information was extracted from the included articles: author, year of publication, country of origin, type of study, objective, sample characterization, relevant considerations, and level of evidence. The results were synthesized according to the similarity of themes and study types. Finally, descriptive statistics were used to analyze the results. The level of evidence and grade of recommendation of the studies were analyzed according to the Joanna Briggs Institute classification (9) as described in Table 2.

**Table 2:**
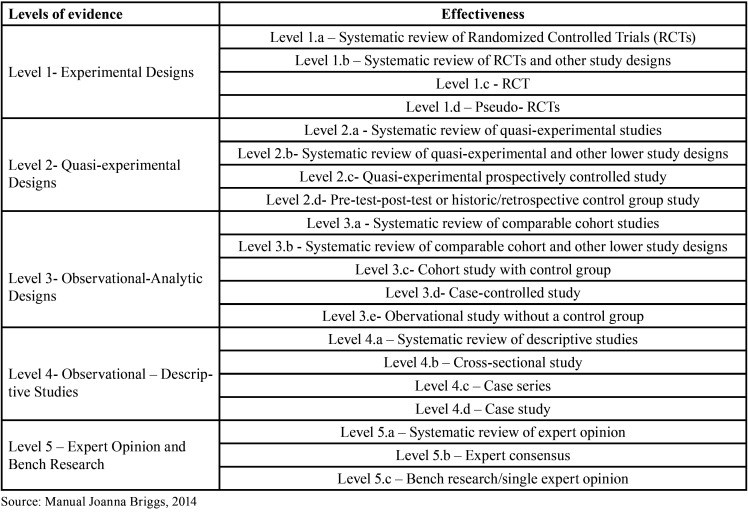
Level of evidence and grade of recommendation of the studies, according to the Joanna Briggs Institute classification (9).

## Results

-Database Search

**Figure 1:**
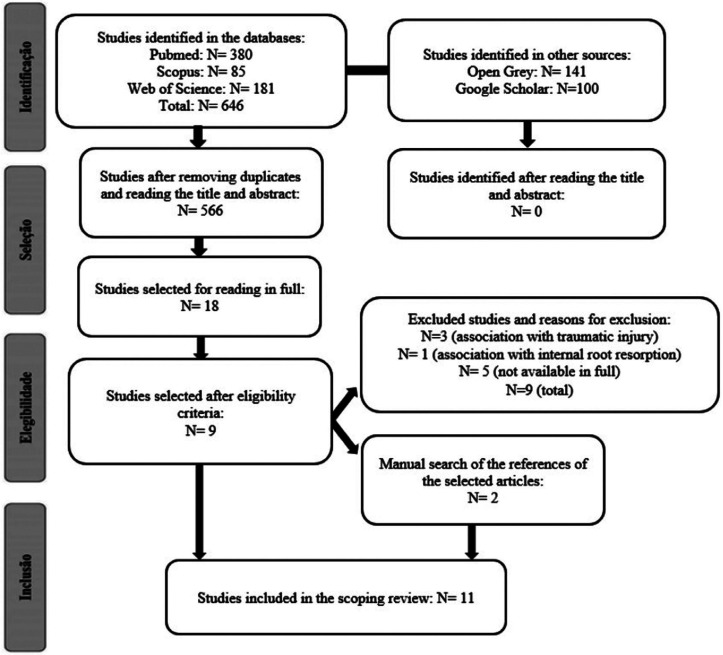
PRISMA Flowchart for study selection.

A total of 646 studies were identified by searching the PubMed, Scopus, and Web of Science databases, and 241 studies in the grey literature. After removing duplicates, using the reference manager EndNote and the individual visual checking, followed by reading the titles and abstracts, 19 articles remained for reading in full. After applying the eligibility criteria and manually searching the references of the articles selected from the databases, 11 studies were included in this scoping review. Figure 1 shows the reason for excluding the studies and more details regarding the selection. Table 3 shows the characterization of the studies along with the relevant considerations.

**Table 3:**
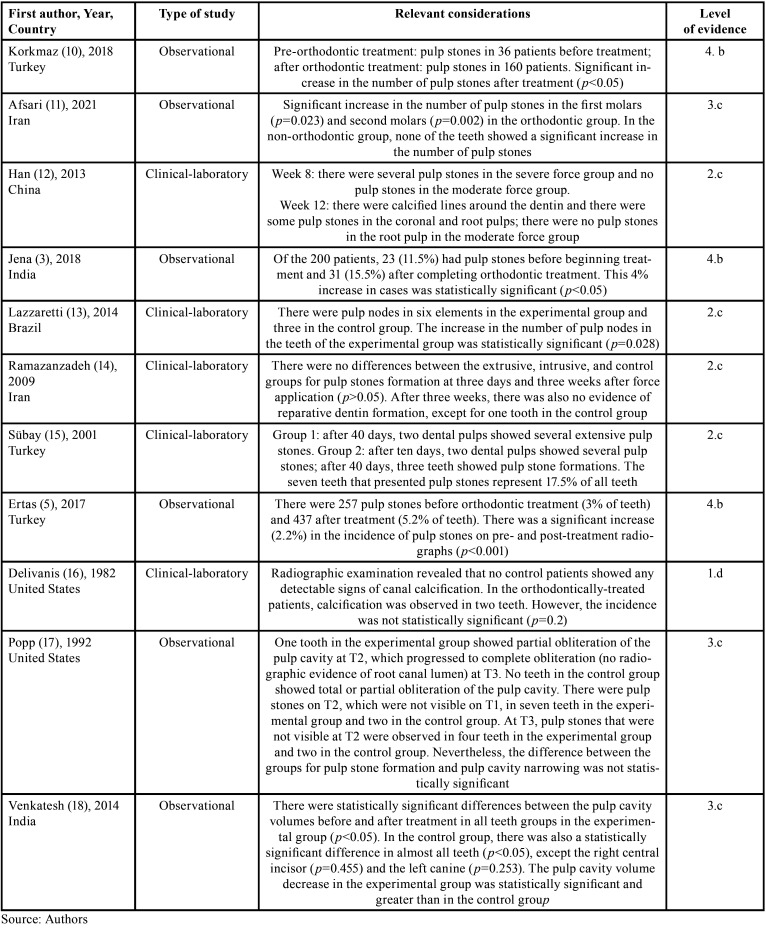
Characteristics of the included studies.

The levels of evidence were categorized into: 4.b - n=3, cross-sectional studies;3, 5, 10 2.c - n=4, prospective controlled quasi-experimental study;12, 13, 14, 15 1.d - n=1, pseudo-randomized controlled trial;16 and 3.c - n=3, cohort study with control group11, 17, 18 (Table 1).

Bibliometric analysis of included studies

**Figure 2:**
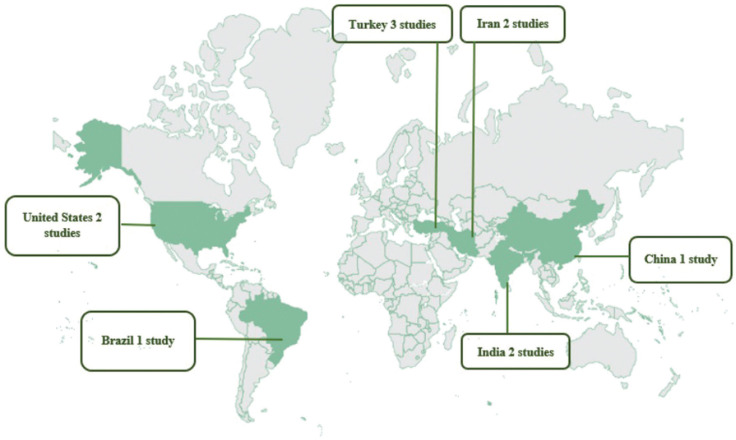
Worldwide distribution of studies selected in the scoping review.

The 11 studies included in this review were concentrated in six countries: one in China, one in Brazil, two in India, two in Iran, two in the United States, and three in Turkey. The sample size for the included observational and experimental studies amounted to 1817 subjects, ranging from 13 to 545 participants (Fig. 2).

-Presence of pulp stones and decreased pulp cavity

Four observational design studies included in this review showed a statistically significant increase in the number of pulp stones after orthodontic treatment. These studies included: Ertas *et al*. (5) (2017) - *p*<0.001; Jena *et al*. (3) (2018) - *p*<0.05; Korkmaz *et al*.10 (2018) - *p*<0.05; and Afsari *et al*. (11) (2021) - *p*<0.05.

In the observational study by Venkatesh *et al*. (18) (2014), there were statistically significant differences between pulp cavity volumes in all teeth in the experimental group (*p*<0.05) and most teeth in the control group, except for the right central incisor (*p*=0.455) and the left canine (*p*=0.253). Thus, they observed a greater volume decrease in the experimental group. In Popp *et al*. (17) (1992), there was no statistically significant difference between the experimental and control groups for both pulp stone formation and pulp cavity narrowing.

In clinical-laboratory studies, Han *et al*. (12) (2013) noted that several pulp stones were formed after eight and 12 weeks of severe orthodontic force application. However, the formation of calcified lines was observed around the dentin only after 12 weeks. Lazzaretti *et al*. (13) (2014) and Sübay *et al*. (15) (2001) observed a statistically significant increase in pulp stones in their experimental groups compared to controls, with the former showing a *p-value* of 0.028. In the latter, pulp stone formation accounted for 17.5% of all teeth.

In Ramazanzadeh *et al*. (14) (2009), pulp stone formation was not statistically significant between the control group and the groups in which intrusive and extrusive forces were applied. Furthermore, there was no formation of reparative dentin in the experimental groups. Delivanis *et al*. (16) (1982) observed that in the control group, in which no orthodontic forces were exerted, the presence of pulp calcification was not identified. Meanwhile, pulp calcification was evidenced in two teeth in the group of orthodontically treated patients. However, statistical analysis reveals that the incidence was not significant.

## Discussion

This scoping review mapped the scientific evidence in the literature regarding the association of orthodontic treatment and pulpal calcifications, bringing as main results the increased frequency of pulp stones (3,5,10-13,15,16,18) and decreased root canal lumen, in addition to a reduction in pulp cavity volume (12,14,16,18). These results corroborate the findings of other studies, which state that pulpal calcifications seem to increase in number and size in patients undergoing orthodontic treatment (19-22).

Many factors can influence orthodontic mechanics, such as type of movement, force dimension, the mechanical method used, and the patient’s age, periapical condition, treatment time, and traumas. In addition, orthodontic forces can produce periodontal inflammatory reactions but should not lead to severe dental pulp inflammation (23). When these changes occur in the pulp tissue, there may be a decrease in the pulp chamber and root canal lumen through the formation of secondary dentin, the formation of pulpal calcifications, and even pulpal necrosis (4,13). Since the methodologies of the studies included in this review bring distinct designs, as well as orthodontic movements and forces also very varied, we suggest more studies, either observational or experimental, controlled so that the syntheses of evidence regarding the topic can contribute in clinical decisions by professionals.

All studies included in this review report the presence of pulpal calcifications or pulp nodes after orthodontic treatment, with six significantly increasing this change (3,5,10-13). Therefore, it should be considered that the relationship between orthodontic forces and pulp nodes is not uncommon (11,13) as also identified in Vitali *et al*. (19) (2022), which observed an increase in the presence of pulp nodes from 1.56% to 6.5% after fixed orthodontic treatment. In another review (20) conducted by the same author, three of the 31 studies showed an increase in pulp nodes after orthodontic force application.

Ramazanzadeh *et al*. (14) (2009), Sübay *et al*. (15) (2001), Delivanis *et al*. (16) (1982), and Popp *et al*. (17) (1992) found no significant difference in the increase in pulp nodes after orthodontic treatment. In Ramazanzadeh *et al*. (14) (2009), the force was applied for a reduced time of three days and three weeks, so it may not have been able to cause calcification formation. Sübay *et al*. (15) (2001), in addition to a similarly short time frame, had a small sample of participants. The studies by Delivanis *et al*. (16) (1982) and Popp *et al*. (17) (1992) have older methodologies. In Delivanis *et al*. (16) (1982), the authors report that the observation was based on available periapical radiographs, not detailing which teeth were analyzed or whether these radiographs were performed in a standard manner in all patients, which may have interfered with the finding of calcifications. In Popp *et al*. (17) (1992), the radiographic evaluation included only incisors, which may be related to the low number of calcifications since the most affected teeth were, in descending order, the molars, followed by premolars and lastly, the incisors (24).

The dental pulp reacts early to orthodontic movement through vascular and circulatory changes (25). Furthermore, it is a histologically noticeable alteration in the layer of odontoblasts, connective edema, and an increase in progenitor cells and fibroblasts, evidencing an adaptive inflammatory reaction to the forces applied (26,27). The deposition of secondary dentin is a consequence of this reaction, which may interfere with pulp cavity dimensions, reducing the pulp chamber and root canal lumen of teeth submitted to orthodontic treatments (28).

This review’s results show a gap in the number of studies that specifically portray the outcome of pulp calcification because of the increased frequency of decreased root canal lumen and reduced volume of the pulp cavity due to dentin deposition. Only five studies bring this topic (12,14,16-18), which is in line with the recent systematic review by Vitali *et al*. (19) (2022). They reported a significant increase in pulp cavity narrowing after five years of fixed orthodontic treatment and a decrease in root canal lumen. After 17-18 months of treatment, there was a reduction in pulp cavity volume.

Han *et al*. (12) (2013) report that the group in which severe orthodontic forces were applied for 12 weeks showed calcified lines around the dentin. Venkatesh *et al*. (18) (2014) noted that the pulp cavity volume reduction in the experimental group was statistically significant and greater than in the control group. Delivanis *et al*. (16) (1982), even though they did not find a statistically significant result, noted that of 46 patients treated with orthodontics, two teeth showed calcification. Ramazanzadeh *et al*. (14) (2009) found no evidence of reparative dentin formation after three weeks of the force application. Thus, there was no statistically significant result. In this study, it is worth noting that the time of force application was short, which may have interfered with the results. In Popp *et al*. (17) (1992), the study included adolescent patients with a follow-up of more than five years. The authors reported that the narrowing of the pulp canals in both the experimental and control groups, without a significant difference, may have been observed due to the natural aging process.

The main morphological changes of calcifications include pulp stones and diffuse calcifications (29). Usually, pulp stones are more frequently located in the pulp chamber, while calcifications are more diffuse and more prevalent in root canals (30). In both cases, there may be repercussions on dental procedures, especially in endodontics, entailing a challenge for clinicians and specialists because they hinder access to root canals and subsequent chemical and mechanical cleaning (31,32), which may cause failure in endodontic treatment or even tooth loss, directly affecting oral health and quality of life.

The identified limitations include a great variation in the designs and methodologies employed, a reduced number of studies, lack of standardization of movement type, force, and time, among other variables. Furthermore, most studies showed low evidence, stressing the low production on the topic, which justifies the demand for future research. Another important issue to be highlighted concerns the worldwide distribution of the selected studies, limited to a few countries, as shown in Figure 2. Thus, we recommend a broader approach, with participants from other continents, because it would provide more confidence to the professionals regarding clinical procedures.

## Conclusions

Regardless of the abovementioned limitations, this review has the potential to support new investigations, mapping out gaps in studies that relate pulpal calcifications to orthodontic treatment and encouraging more robust clinical research, such as randomized clinical trials, providing grounds for clinical decision-making.

The importance of the theme addressed in this review is evident because, although pulpal calcifications do not cause major repercussions in healthy teeth, much attention should be devoted to teeth requiring dental interventions since they may cause problems during treatment. In these cases, calcifications must be identified, and a good treatment plan must be devised, especially if endodontic treatment is required.
